# Scedosporium apiospermum Pneumonia in an Immunocompetent Host

**DOI:** 10.7759/cureus.16891

**Published:** 2021-08-04

**Authors:** Wasey Ali Yadullahi Mir, Dhan B Shrestha, Mahammed Z Khan Suheb, Shravani Reddy, Suman Gaire

**Affiliations:** 1 Department of Internal Medicine, Mount Sinai Hospital, Chicago, USA; 2 Department of Medicine, Mount Sinai Hospital, Chicago, USA; 3 Critical Care, AdventHealth, Orlando, USA; 4 Department of Internal Medicine, Rush University Hospital, Chicago, USA; 5 Department of Emergency Medicine, Palpa Hospital, Palpa, NPL

**Keywords:** scedosporium, scedosporiosis, invasive fungal infections, immunocompromised host, pneumonia, bronchiectasis

## Abstract

Invasive fungal infections are being increasingly identified recently. *Scedosporium* is a significant cause of non-Aspergillus mold infection. It can cause disseminated disease in an immunocompromised host and localized pulmonary infection in immunocompetent ones, especially in those with preformed lung cavities. We present a case of scedosporiosis in an elderly female with bronchiectasis who presented with refractory pulmonary symptoms and infiltrates. The case emphasizes the need to keep the fungal infection in the differential diagnosis of refractory infiltrates in immunocompetent individuals without preformed cavities if they have bronchiectasis. Voriconazole monotherapy can be used as the first-line in proven cases of scedosporiosis.

## Introduction

Although aspergillosis is the most common cause of invasive mold infection, other causes are also increasingly identified [1]. Among causes of non-Aspergillus mold infection, *Scedosporium* is one of the major causes of invasive fungal infection [1,2]. Invasive fungal infections can present with a diverse range of clinical presentations. They can cause pulmonary infection, disseminated skin, soft tissue, sinus, and brain infection in an immunocompromised host. The most important species causing scedosporiosis is *Scedosporium apiospermum. Scedosporium* species are widespread in cattle dung, sewage, polluted water, oil-soaked lands, agricultural land, urban playgrounds, and industrial areas [3,4]. Scedosporiosis is of particular clinical interest as it is highly resistant to many antifungal agents.

We present a rare case of invasive mold (*Scedosporium apiospermum*) pneumonia in an immunocompetent host with preexisting lung disease.

## Case presentation

An 83-year-old female presented with complaints of shortness of breath, cough with blood-tinged sputum, and fatigue for the past several months. She was on warfarin for chronic atrial fibrillation and 2L/min home oxygen for chronic obstructive pulmonary disease (COPD). Her medical history was also significant for hypertension. She denied a history of smoking. At presentation, she was hypoxemic with a history of domiciliary oxygen use at 2L/min flow. Otherwise, she had normal vital parameters. On physical examination, coarse breath sounds were heard in her lower lung lobes bilaterally.

Her blood routine revealed mild leukocytosis of 12,000 with neutrophilic predominance (80%). Computed tomogram (CT) chest showed mucus plugging, bronchiectasis with tree-in-bud infiltrates at the lower lobe level without any cavitary lesions. Initial sputum culture grew *Proteus mirabilis*, so she was transitioned from broad-spectrum vancomycin and piperacillin/tazobactam to levofloxacin. Acid-fast bacilli (AFB) sputum, AFB culture, and legionella urine antigen were all negative. An echocardiogram showed increased pulmonary artery pressure (61 mmHg). She was treated with a tapering dose of prednisone, antibiotics and discharged on 2L home oxygen.

Following discharge, she had minor improvement in dyspnea and hypoxia. Later her dyspnea worsened, requiring multiple courses of prednisone taper and doxycycline for presumed COPD exacerbation. Over time, her symptoms did not improve. Therefore, for refractory dense lower lobe infiltrates with bronchiectasis, further investigations were ordered. Serologies for Histoplasma, Blastomyces, coccidioidomycosis, *Aspergillus fumigatus* IgG and (1,3)-beta-D-glucan and all allergy testing were negative.

Bronchoscopy was notable for bilateral mucoid secretions, and a left lower lobe anterior segment bronchoalveolar lavage (BAL) sample was negative for acid-fast smear, *Mycobacterium tuberculosis *polymerase chain reaction (PCR), and galactomannan. However, at this time, (1,3)-beta-D-glucan was greater than 500, and the fungal culture from lavage grew the *Scedosporium apiospermum* complex. In addition, BAL cytology showed rare acute angle branching septate hyphae suggestive of *Aspergillus.* Subsequently, sputum culture also grew *Scedosporium apiospermum.*

She was successfully treated with a standard dose of voriconazole, i.e., 6mg/Kg intravenously twice a day for the first 24 hours, followed by 4mg/Kg twice-daily dosing. At the time of discharge to home, the patient was kept on voriconazole 200mg twice daily per oral dosing and kept on regular follow-up. Warfarin was switched to apixaban owing to warfarin’s interaction with voriconazole. Her dyspnea significantly improved after six months of therapy, and she was successfully weaned off oxygen as well.

## Discussion

Although* Scedosporium* infection is more commonly seen with immunocompromised patients, it can also cause disease in immunocompetent hosts [5-7]. It is the second most common fungal infection in cystic fibrosis patients after *Aspergillus* [8]. In immunocompromised hosts, it causes necrotizing pneumonia, central nervous system infection, ocular infection, and endocarditis [6,7]. In immunocompetent hosts, *Scedosporium* causes traumatic local skin, soft tissue, joint infections, and pulmonary infection primarily due to colonization in a preformed cavity. The upper and lower respiratory tract are the most commonly involved sites in non-immunocompromised hosts. The respiratory involvement can fall into different categories: transient local colonization, bronchopulmonary saprobic involvement, fungus ball formation, and invasive pseudallescheriasis (*Pseudallescheria pneumonia*). *Scedosporium* can also present clinically similar to allergic bronchopulmonary aspergillosis [8].

Scedosporiosis can be diagnosed with histopathology, fungal culture, or by identification of fungal DNA with a polymerase chain reaction. In the microscopic examination, the hyphae are seen similar in all molds, making it hard to distinguish scedosporiosis from other invasive septate molds [9]. Since *Aspergillus* is more common, the cytology findings were suggestive of *Aspergillus*. However, the culture could distinguish among them and provide the definitive diagnosis.

Medical management of scedosporiosis is challenging because it is resistant to many antifungals, and the disease tends to relapse. *Scdesporium apiospermum* is resistant to fluconazole, ketoconazole, flucytosine and terbinafine and is generally sensitive to voriconazole and itraconazole [10]. Therefore, a sensitivity study with minimum inhibitory concentration should be done in all diagnosed cases with scedosporiosis. Voriconazole has proven efficacy in treating scedosporiosis in multiple studies [11,12]. Surgical debridement is an option for the patients whenever the source of infection is surgically accessible.

The prognosis of scedosporiosis is very poor. However, the prognosis seems to be better in cases of infection in immunocompetent patients. In a meta-analysis of scedosporiosis in immunocompetent individuals, the mortality was 12.5% [13].

Although being immunocompetent, bronchiectasis increased the susceptibility to scedosporiosis in our patients. In the metanalysis, bronchiectasis was the second most common cause of scedosporiosis in immunocompetent individuals after pulmonary tuberculosis [13]. As fungal infections are rare in immunocompetent individuals, especially those who don’t have a preformed lung cavity, it does not come high up in differential and is diagnosed late. Our patient responded well to voriconazole and recovered completely.

## Conclusions

This case emphasizes that chronic respiratory failure and refractory infiltrates need further investigation for atypical organisms, including fungal etiologies. This remains particularly helpful in the case of long-standing bronchiectasis, as in our patient, where there might only be dilatation of airways but no distinct cavities. This immunocompetent host might have acquired rare opportunistic mold infection from the gardening hobby in preexisting structural lung disease. *Scedosporium* species pose a therapeutic challenge due to their intrinsic resistance to antifungal agents and their tendency to relapse despite their susceptibility to the treatment. Voriconazole monotherapy should be continued until the resolution of symptoms.
